# Molecular Characterization of *Echinococcus granulosus sensu lato* from Humans in Slovenia

**DOI:** 10.3390/pathogens9070562

**Published:** 2020-07-12

**Authors:** Barbara Šoba, Špela Gašperšič, Darja Keše, Tadeja Kotar

**Affiliations:** 1 Institute of Microbiology and Immunology, Faculty of Medicine, University of Ljubljana, Zaloška 4, 1000 Ljubljana, Slovenia; gaspersicspela@gmail.com (Š.G.); Darja.Kese@mf.uni-lj.si (D.K.); 2 Department of Infectious Diseases, University Medical Centre Ljubljana, Japljeva 2, 1000 Ljubljana, Slovenia; tadeja.kotar@kclj.si

**Keywords:** *Echinococcus granulosus sensu lato*, *Echinococcus granulosus sensu stricto*, *Echinococcus canadensis* G7, human infection, Slovenia, molecular characterization

## Abstract

The larval form of tapeworms of the *Echinococcus granulosus sensu lato* species cluster cause an important zoonotic infection, cystic echinococcosis (CE). Molecular characterization of the cluster’s isolates from different hosts greatly contributes to a better understanding of its transmission dynamics. To date, no genetic information is available on CE in Slovenia. In this work, we characterized isolates from human CE cases. Parasite samples from 18 patients were collected, together with the patients’ demographic and clinical data. Genomic DNA was analyzed by conventional PCR and sequencing at four mitochondrial loci (cytochrome c oxidase subunit 1, *cox1*; NADH dehydrogenase subunit 1, *nad1*; NADH dehydrogenase subunit 5, *nad5*; and small ribosomal RNA, *rrnS*). Thirteen isolates were successfully amplified and sequenced. Seven (58.8%) patients were infected with *E. granulosus sensu stricto* (*s.s.*) G1, five (38.5%) with *E. canadensis* G7 and one (7.7%) with *E. granulosus s.s.* G3. *Echinococcus canadensis* G7, the pig genotype, was identified exclusively in autochthonous Slovenes, while the patients originating from the Western Balkans were all infected with *E. granulosus s.s.* Our findings suggest that pigs are important intermediate hosts for human CE in Slovenia.

## 1. Introduction

Cystic echinococcosis (CE) is a helminthic zoonosis of worldwide distribution caused by the larval form of the tapeworm species cluster *Echinococcus granulosus sensu lato* (*s.l.*). The life cycle of *E. granulosus s.l.* involves dogs and other canids as definitive hosts of the adult form of the parasite, and a herbivorous or omnivorous intermediate host in which the larval form or CE cyst develops [1]. Humans are accidental intermediate hosts and are infected by ingestion of tapeworm eggs shed in the feces of a definitive host. The infection may remain asymptomatic for years before the cysts, primarily located in the liver and lungs, grow large enough to cause symptoms by exerting pressure on adjacent tissue or upon rupture [2]. By the criteria of the Food and Agriculture Organization of the United Nations and World Health Organization, *E. granulosus s.l.* is ranked second in the global ranking of the most important foodborne parasites [3]. Its high public health relevance and significant contribution to disease burden have also been recognized by the European ranking, in which *E. granulosus s.l.* is among the top four foodborne parasites [4]. 

Within the *E. granulosus s.l.* species cluster, a number of species with phenotypic differences in morphology and biology have been identified [1]. It has been confirmed by molecular-based studies that these species are genetically distinct and, on the basis of genetic differences in mitochondrial genes, comprise eight different genotypes [5,6,7,8]. To date, the *E. granulosus s.l.* species cluster has been shown to consist of *E. granulosus sensu stricto* (*s.s.*) (genotypes G1 and G3), *E. equinus* (genotype G4), *E. ortleppi* (genotype G5), *E. canadensis* cluster (genotypes G6-8 and G10) and *E. felidis* [9,10,11,12,13]. 

*Echinococcus granulosus s.s.* genotype G1 has the most cosmopolitan distribution and is responsible for the great majority (almost 90%) of human CE. Its principal intermediate host appears to be sheep [14,15]. Although considerably fewer (7%) infections have been attributed to *E. canadensis* genotype G6 than to *E. granulosus s.s.* genotype G1, the former genotype was found to be the second most common cause of human CE worldwide [14]. *Echinococcus canadensis* genotype G7 appears to be largely limited to certain areas in Central and Eastern Europe, with pigs as the most common intermediate host. It has been responsible for about 4% of human CE cases [14]. Human infections with genotypes G5, G8 and G10 are rare and no cases of human CE caused by G4 have been described [14,15]. 

In Slovenia, echinococcosis in humans is included in the list of notifiable diseases, and cases are reported to the European Centre for Disease Control. The mean annual incidence rate of the disease in the period between 2007 and 2016 was 0.30 per 100,000 inhabitants, slightly higher than the European value for the same period, which was 0.20 per 100,000 inhabitants [16,17]. To date, no genetic information is available on the *E. granulosus s.l.* species and genotypes circulating in Slovenia. The main goal of the present study was therefore to characterize at the molecular level human isolates of *E. granulosus s.l.* collected in Slovenia using established molecular methods. 

## 2. Results

Cyst fluids were obtained from 18 CE patients. Demographic and clinical data were available for 13 of them (Table 1). Of these, seven were men and six were women. The mean age of these patients was 42.5 years (min. 9 years, max. 74 years). In all patients, cysts were located in the liver. One patient (SI12-H8) was reported to have secondary CE due to dispersion from the previous intervention of pulmonary CE eighteen years before. All other patients suffered from primary CE. Liver cysts from the patients were small (<5 cm; 30.8%), medium (5–10 cm; 53.8%) or large (>10 cm; 15.4%) in size. Only three cysts were classified according to the World Health Organization Informal Working Group on Echinococcosis (WHO-IWGE) classification criteria. SI19-H16 and SI19-17 were classified as CE4 and SI20-18 as CE3b/CE4. Before puncture, aspiration, the injection of protoscolicide and reaspiration (PAIR) or surgery, sera from seven (53.8%) of 13 patients tested positive by indirect hemagglutination assay and/or Western blot, while sera from six (46.2%) patients tested negative. By the time of the study, all patients had recovered from CE and their ongoing follow-up involves regular medical checkups.

Thirteen (72.2%) out of the 18 human isolates were successfully amplified and sequenced at least at two loci tested. Sequence data were generated from the four loci (*cox1, nad1, nad5, rrnS*-1) for 11 out of 12 (91.7%) human cyst fluids, which were stored at −20 °C, while the amplification and sequencing of one human cyst fluid (SI16-H12) stored at −20 °C were successful only at two loci (*nad5, rrnS*-2). Of the isolates from six human cyst fluids that were preserved in formalin, only one (16.7%) was successfully genotyped—its amplification and sequencing were successful at three loci (*cox1, nad5, rrnS*-2).

**Figure 1:**
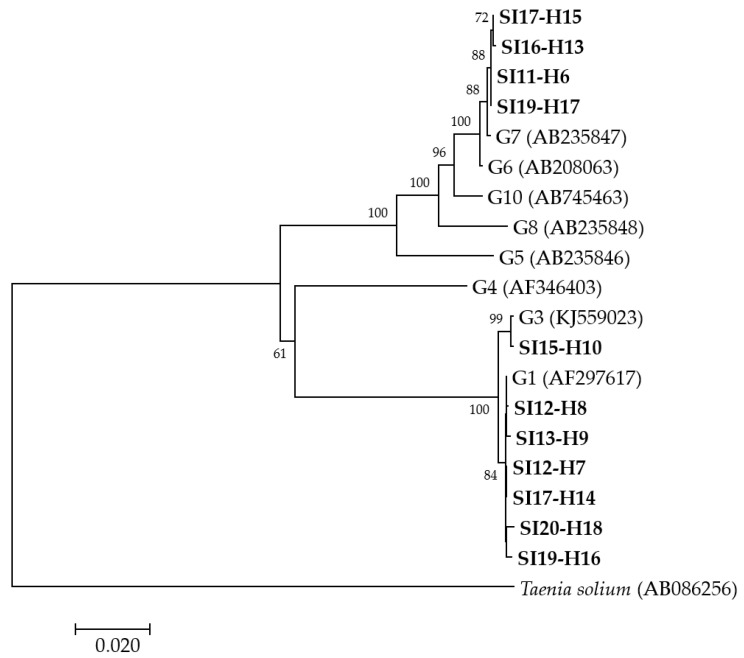
Neighbor-joining phylogenetic tree of concatenated *cox1, nad1, nad5* and *rrnS*-1 partial mitochondrial gene sequences of *Echinococcus granulosus sensu lato* isolates from humans examined in the present study and representative sequences retrieved from GenBank. Isolates examined in the present study appear in boldface. Values on branches are percentage bootstrap values using 1000 replicates. Bootstrap values greater than 50% are shown.

A phylogenetic analysis was performed from concatenated *cox1, nad1, nad5* and *rrnS*-1 partial mitochondrial gene sequences for 11 human isolates (Figure 1) revealing that, of these, six isolates grouped with *E. granulosus s.s.* G1, one with *E. granulosus s.s.* G3 and four with *E. canadensis* G7. Since the amplification and sequencing of two human (SI-H4 and SI16-H12) isolates were not successful at all of the four loci, another phylogenetic analysis was performed from concatenated *nad5* and *rrnS*-2 partial mitochondrial gene sequences (data not shown). The analysis revealed that the isolates SI-H4 and SI16-H12 grouped with *E. granulosus s.s.* G1 and *E. canadensis* G7, respectively. The groupings of the other isolates remained the same as in the first analysis (data not shown). 

Altogether, the molecular characterization revealed that of the 13 humans whose isolates were successfully genotyped, seven (58.8%) were infected with *E. granulosus s.s.* G1, one (7.7%) with *E. granulosus s.s.* G3 and five (38.5%) with *E. canadensis* G7 (Table 1). 

All five isolates from autochthonous Slovene CE patients were identified as *E. canadensis* G7. *Echinococcus granulosus s.s.* G1 was identified in two immigrants from North Macedonia, three patients originating from Bosnia and Herzegovina, a patient of Albanian ethnicity, and one patient whose demographic data were not available. *Echinococcus granulosus* G3 was identified in only one patient, whose country of origin was Bosnia and Herzegovina. 

## 3. Discussion

The results of the present study offer the first insight into *E. granulosus s.l.* genetic diversity in humans in Slovenia. Three genotypes were detected: *E. granulosus s.s.* genotypes G1 and G3 and *E. canadensis* genotype G7.

As is evident from Table 1, the CE patients from our study can be divided into two groups depending on their origin; the first group represented autochthonous Slovenes and the second patients who had immigrated to Slovenia from Western Balkan countries. It is well known that socio-cultural factors and thus human behavior contribute to the epidemiology and transmission dynamics of zoonoses, including echinococcosis [18,19,20]. Eating habits may have an influence on the livestock spectrum in an area and consequently contribute to the genetic makeup of zoonotic pathogens. In the present study, CE patients originating from Western Balkan countries were all infected with *E. granulosus s.s.*, while the pig genotype *E. canadensis* G7 was identified exclusively in autochthonous Slovenes (Table 1). The differences in the causative agents of human CE in the two patient groups may reflect their socio-cultural specifics. It is noteworthy that *E. canadensis* genotype G7 is the major causative agent of human CE in the autochthonous population from neighboring Austria, other genotypes being almost exclusively related to travel or non-Austrian origin [21]. In the Austrian study [21], Schneider et al. reported that 92% of 25 CE patients of Austrian origin were infected with genotype G7, indicating this genotype’s importance in human CE in Central Europe. Of other European countries, genotype G7 is the predominant human CE agent in Eastern Europe: in particular, in Poland, the Slovak Republic and the Baltic countries [22,23,24,25,26,27,28]. 

Interestingly, it was already hypothesized back in the 1970s that pig is the most important intermediate host in the epidemiological and epizootiological conditions of CE in Slovenia [29]. Since then, the annual incidence of human echinococcosis has decreased significantly, due to controlled slaughtering, the better general and sanitary attitude of livestock breeders, and the deworming of dogs, but CE transmission is still ongoing [30]. It appears that pigs are indeed important intermediate hosts for human CE in Slovenia, which is supported by our identification of *E. canadensis* genotype G7 in autochthonous Slovenes. Echinococcosis in pigs is recorded by the Administration of the Republic of Slovenia for Food Safety, Veterinary and Plant Protection practically every year, although prevalence values are very low (less than 0.1%) [31]. We assume that home slaughtering, which is allowed only for consumption by a family, may be an important risk factor for transmission and a prerequisite for the perpetuation of the dog-pig lifecycle. Such a hypothesis had been evoked in other regions of Europe, e.g., Corsica, where free-ranging practices and home slaughtering may explain the presence of *E. canadensis* G7 in pigs [32], and Lithuania, where *E. canadensis* G7 was the only genotype identified in pigs and humans [22,26]. 

As mentioned above, of the successfully genotyped cysts from the patients from the Western Balkans, all were characterized as *E. granulosus s.s.* Two genotypes were identified: one patient was infected with genotype G3 while the other six were infected with genotype G1. With its wide host range, *E. granulosus s.s.* accounts for most of the global burden of CE in humans and livestock and also represents the principle causative agent of CE in southeastern Central Europe [14,15]. In our study, more than half of the patients from the Western Balkans originated from Bosnia and Herzegovina, a former Yugoslav republic in which more than 50% of the population is Muslim and, due to Islamic dietary habits, does not eat pork but primarily lamb, beef and poultry. The identification of *E. granulosus s.s.* rather than pig genotype *E. canadensis* G7 in the patients from Bosnia and Herzegovina is therefore not surprising. Genotype G1 was also detected in patients from Bosnia and Herzegovina in the study of Schneider et al. [21], while patients from the same study originating from the former Yugoslav republics Serbia and North Macedonia were infected with *E. granulosus s.s.* G1 and *E. canadensis* G7 [21]. Two patients of North Macedonian origin from our study were both infected with *E. granulosus s.s.* G1. Since immigrants to Slovenia predominantly originate from ex-Yugoslav republics, we searched for English-language publications on *Echinococcus granulosus s.l.* genotypes circulating in livestock, dogs and humans in those geographical regions. In Serbia, *E. granulosus s.s.* has been detected in sheep, while *E. granulosus s.s.* G1 and *E. canadensis* G7 have been identified in humans, cattle and pigs [15,33,34]. Infections with *E. granulosus s.s.* have been characterized in dogs and cattle in Kosovo [15,35]. Apart from the study of Schneider et al. [21], there are no data on the genetic makeup of *Echinococcus granulosus s.l.* in humans from Bosnia and Herzegovina or North Macedonia. To the best of our knowledge, there are also no data on *Echinococcus* genotypes infecting livestock and dogs in these two countries, nor humans and animals in Croatia or Montenegro.

Although formerly known as the “buffalo strain”, *E. granulosus s.s.* G3 has a wide range of intermediate hosts and is now recognized to circulate in almost all continents [15,36]. It is significantly less prevalent worldwide than genotype G1 but was found to be associated with a high rate of human CE cases in Italy, Bulgaria, Pakistan, Iran and India [37,38,39,40,41]. Until recently, it was not possible to differentiate unambiguously between genotypes G1 and G3, so the real contribution of genotype G3 to the global burden of CE may have been underestimated. Several recent publications have outlined the advantages of using long sequences of mitochondrial DNA in the molecular analysis of *E. granulosus s.s.*, providing a reliable method of discriminating G1 and G3 from each other [7,36,42]. It seems that the best target for the identification of G1 and G3 is the *nad5* gene region [43]. In our study, genotype G3 was identified in an immigrant from Bosnia and Herzegovina, which adds to knowledge of this genotype’s wide geographic distribution.

Regarding the clinical presentation of CE, it has been speculated that *E. canadensis* G7 cysts are smaller and might be symptomless for decades and, consequently, identified at an older age [21,44]. In the study of Schneider et al. [21], the diagnosis of the *E. canadensis* G7-infected patients occurred more often incidentally—meaning that the patients were more often asymptomatic at the time of the diagnosis—than patients infected with *E. granulosus s.s*. Unfortunately, the number of cysts in our study was low, which makes it impossible to verify these hypotheses. 

Immunodiagnostic procedures for serum antibody detection have a confirmatory role in the diagnosis of human CE. Serology should not be used alone but always in conjunction with imaging techniques [44,45]. One of the drawbacks of serological methods is their variable sensitivity, which may range from 54% to 100% and depends on several factors: cyst location, cyst stage, time of serum collection, number and size of cysts, tested population, etc. Additionally, different genotypes of the *E. granulosus s.l.* species cluster potentially express different antigenic sets, which may lead to the different diagnostic performance of serological tests [46]. In our study, of the CE patients whose serological data were available, 46.2% tested negative by indirect hemagglutination assay and/or Western blot before surgery or PAIR. Since only three cysts from the present study were classified according to the WHO-IWGE classification criteria, we cannot comment on the effect of the cyst stage on serology results. However, it was demonstrated in previous studies that false-negative serology results may often occur in cases of CE with young or inactive cysts [45].

In our study, light microscopy examination of the cyst fluids allowed for the assessment of protoscoleces and/or hooks in all samples, verifying that the cysts were fertile. As measured by spectrophotometer, the DNA concentration and purity in all DNA isolates were adequate. However, out of the six formalin-preserved samples, five showed no amplification at any locus. It is well known that formalin can cause DNA fragmentation, resulting in a significant reduction of the amplifiable templates, which increases with longer storage time [47]. Nevertheless, due to the low number of available cysts, the formalin-preserved material was an invaluable source for our study: one formalin-preserved sample (SI-H4) was successfully amplified, showing amplicons at three loci. Several molecular studies of the *Echinococcus granulosus s.l.* species cluster in formalin-fixed samples have encountered the same problem that we had. The authors of these studies reported that between 8% and 58% of such samples were not amplified successfully [48,49,50,51]. DNA extraction and amplification from formalin-fixed samples is generally problematic but recent improvements in DNA extraction methods and the amplification of short fragments of the target DNA enhanced the detection and identification of infectious agents in these samples [52]. In accordance with this, we tried to adapt our PCR strategy to samples with degraded DNA by amplifying a shorter fragment (approx. 285 bp) of the *rrnS* gene. Indeed, the amplification of the above-mentioned sample SI-H4 at the *rrnS* locus was successful only when a shorter fragment of this gene was amplified. However, as discussed in previous studies, the use of a target size shorter than 200 bp might have increased the sensitivity even further [52,53]. An interesting approach was described in the study of Koonmee et al. [52], where repeated PCR using the same primer sets and conditions was successfully employed for the amplification of short DNA fragments from formalin-fixed paraffin-embedded (FFPE) tissues. It was proposed by the authors that the method could be applied for FFPE tissues in general [52].

## 4. Materials and Methods 

### 4.1. Human Samples

For diagnostic purposes, 18 cyst fluids from 18 CE patients were referred to the Laboratory for Parasitology at the Institute of Microbiology and Immunology (IMI), Faculty of Medicine, University of Ljubljana, Slovenia, up to February 2020. All cysts were identified as fertile (presence of protoscoleces and/or hooks) upon light microscopy examination. Thirteen cyst fluids were obtained between 2011 and 2020, while five were collected before 2011. The material was either stored unpreserved at −20 °C or preserved in 10% formalin and stored at +4 °C until molecular analysis (Table 1). According to the principles expressed in the Declaration of Helsinki, the Oviedo Convention on Human Rights and Biomedicine, and the Slovene Code of Medical Deontology, all human samples were anonymized and data on patient gender, age and region of origin were linked only to randomized numerical codes. The demographic and clinical data for the 13 patients whose cyst fluids were obtained between 2011 and 2020 are presented in Table 1. For these patients, the results of their serological testing for the presence of anti-Echinococcus IgG antibodies by indirect hemagglutination assay (Cellognost Echinococcosis IHA, Siemens Healthcare Diagnostics, Marburg, Germany; cut-off titer 1:32) and confirmatory Western blot (Echinococcus WB IgG, LDBIO Diagnostics, Lyon, France) are also available (Table 1). No demographic and clinical data are available for the five patients whose samples were obtained before 2011.

### 4.2. Molecular Analyses

Prior to DNA extraction, cyst fluids were centrifuged at 3500 g for 5 min, the formalin, if added, was removed, and the pellets were washed three times for 45 min in phosphate-buffered saline (PBS). After washing, all samples were centrifuged at 3500 g for 5 min and the PBS was removed. Genomic DNA was extracted from each sample using a commercial QIAamp DNA Mini Kit (Qiagen, Germany), following the manufacturer’s protocol for DNA purification from tissues. The extracted DNA was checked with NanoDrop 2000 (ThermoFisher Scientific, Waltham, MA, USA) for qualitative and quantitative appropriateness. The DNA extracts were stored at −20 °C until analysis.

Fragments of cytochrome c oxidase subunit 1, *cox1* [38], NADH dehydrogenase subunit 1, *nad1* [38,54], NADH dehydrogenase subunit 5, *nad5* [55] and small ribosomal RNA, *rrnS* [55,56] mitochondrial genes were amplified by conventional PCR as described previously, using FastStart™ High Fidelity PCR System (Roche, Germany) reagents. The sequences of the primers are shown in Table 2. For the amplification of the *nad1* gene fragment, nested PCR was performed. Since a fragmentation of DNA was expected for the samples preserved in 10% formalin, PCR with primers amplifying a shorter fragment (approx. 285 bp) of the *rrnS* gene (*rrnS*-2) [55] was also performed when PCR amplifying a longer fragment (approx. 775 bp) of the *rrnS* gene (*rrnS*-1) [56] was negative. 

PCR products were detected by electrophoresis in 2% agarose gels stained with SYBR^®^ Safe DNA Gel Stain (Invitrogen, Carlsbad, CA, USA). The products were subsequently purified using the enzymes FastAP Thermosensitive Alkaline Phosphatase and Exonuclease I (ThermoFisher Scientific Baltics UAB, Vilnius, Lithuania) and sequenced from both strands on an automated sequencer (ABI3500 Genetic Analyzer, Applied Biosystems, Carlsbad, CA, USA) using the same primers as for the amplification (Table 2). The sequences were edited using CLC Main Workbench 7.9.1 (CLC Bio, Aarhus, Denmark) and compared to available sequences from the GenBank database using the Basic Local Alignment Search Tool (BLAST). The nucleotide sequences of *E. granulosus s.l.* identified in this study were deposited in the GenBank database under accession numbers MT227289–MT227300, MT239114–MT239126, MT239132–MT239142, MT253546–MT253556, MT253561 and MT253562.

Multiple alignments were generated using Clustal X version 2 [57]. For phylogenetic analyses, sequences from the current investigation and representative sequences retrieved from GenBank (*E. granulosus* G1 (AF297617), *E. granulosus* G3 (KJ559023), *E. equinus* G4 (AF346403), *E. ortleppi* G5 (AB235846), *E. canadensis* G6 (AB208063), *E. canadensis* G7 (AB235847), *E. canadensis* G8 (AB235848), *E. canadensis* G10 (AB745463)) were used. The sequences from the loci were concatenated and phylogenetic trees were inferred by neighbor-joining (NJ) methods using the Kimura 2 parameter algorithm, as implemented in the MEGA software, version 7.0 [58]. The robustness of the nodes was tested by bootstrap analysis of 1000 iterations. *Taenia solium* (AB086256) was used as an outgroup control sequence.

### 4.3. Ethical Statement

Cyst fluids were collected for diagnostic purposes as a part of a routine diagnostic procedure. The samples were uniquely of parasite origin. No human DNA was included in the study. The samples were obtained in compliance with the ethical standards of the Institutional Review Board of IMI and the study was conducted following the rules of the Declaration of Helsinki. The patients were informed about all diagnostic/identification procedures. According to the principles expressed in the Declaration of Helsinki, the Oviedo Convention on Human Rights and Biomedicine, and the Slovene Code of Medical Deontology, all human samples were anonymized and data on patient gender, age and region of origin were linked only to randomized numerical codes. Since no additional samples or data were collected, the study was deemed to be low-risk and the need for additional ethical permission from the National Medical Ethics Committee was waived.

## 5. Conclusions

The results of this study provide the first insight into the molecular epidemiology of human CE in Slovenia. The identification of *E. canadensis* G7 in autochthonous Slovenes suggests that pigs are important intermediate hosts for human infection in Slovenia. On the other hand, the patients originating from the Western Balkans were all infected with *E. granulosus s.s.* Although this study was limited by the sample size of cyst isolates and therefore the conclusions need to be confirmed through further studies, the differences in the causative agents of human CE in the two patient groups may reflect their socio-cultural specifics. Molecular characterization of the *E. granulosus s.l.* isolates from different hosts would greatly contribute to a better understanding of their epidemiology and transmission dynamics in Slovenia and may have an important implication for planning effective disease control strategies.

## Figures and Tables

**Table 1:** Demographic and clinical data of patients with cystic echinococcosis together with species and genotype of *Echinococcus granulosus sensu lato.*

Patient/ Sample No.	Gender	Age (Years)	Origin ^1^	Year of Sampling	Storage of Sample	No. of Cysts	Cyst Size (cm)	Clinical Presentation	Therapy	Serology		Species, Genotype
										IHA ^2^	WB ^3^	
SI-H1	/ ^4^	/	/	before 2011	+4 °C formalin	/	/	/	/	/	/	/
SI-H2	/	/	/	before 2011	+4 °C formalin	/	/	/	/	/	/	/
SI-H3	/	/	/	before 2011	+4 °C formalin	/	/	/	/	/	/	/
SI-H4	/	/	/	before 2011	+4 °C formalin	/	/	/	/	/	/	*E. granulosus* G1
SI-H5	/	/	/	before 2011	+4 °C formalin	/	/	/	/	/	/	/
SI11-H6	M ^5^	9	Slovenia	2011	−20 °C	1	3 × 2.2 × 1.7	symptomatic	ALB ^6^/12w ^7^ + PAIR ^8^	1:512	+	*E. canadensis* G7
SI12-H7	F ^9^	28	N Macedonia ^10^	2012	−20 °C	1	10 × 6 × 7	symptomatic	ALB/12w + PAIR	1:64	+	*E. granulosus* G1
SI12-H8	M	41	BiH ^11^	2012	−20 °C	1	12 × 10 × 12	asymptomatic	ALB/8w + PAIR + surgery	1:2048	+	*E. granulosus* G1
SI13-H9	F	46	BiH	2013	−20 °C	1	2.8 × 3	symptomatic	ALB/8w + PAIR	−	−	*E. granulosus* G1
SI15-H10	M	37	BiH	2015	−20 °C	2	7.5 × 5.3	asymptomatic	ALB/8w + PAIR	−	−	*E. granulosus* G3
SI16-H11	F	45	BiH	2016	+4 °C formalin	1	6 × 5 × 4	asymptomatic	ALB/10w + surgery	−	−	/
SI16-H12	F	74	Slovenia	2016	−20 °C	1	4.6 × 5	symptomatic	ALB/12w + PAIR	−	−	*E. canadensis* G7
SI16-H13	F	54	Slovenia	2016	−20 °C	1	6 × 6	asymptomatic	ALB/8w + surgery	1:128	+	*E. canadensis* G7
SI17-H14	M	56	N Macedonia	2017	−20 °C	1	16 × 12.5 × 7.5	symptomatic	ALB/12w+PAIR + surgery	1:64	+	*E. granulosus* G1
SI17-H15	M	64	Slovenia	2017	−20 °C	1	5.4 × 4.2	asymptomatic	ALB/12w + PAIR	−	−	*E. canadensis* G7
SI19-H16	M	29	BiH	2019	−20 °C	1	9 × 7 × 9	symptomatic	ALB/12w + PAIR	NP ^12^	+	*E. granulosus* G1
SI19-H17	F	30	Slovenia	2019	−20 °C	1	5 × 8 × 3	asymptomatic	ALB/12w + PAIR	NP	−	*E. canadensis* G7
SI20-H18	M	40	W Balkans ^13^	2020	−20 °C	1	5 × 3.2 × 3	symptomatic	ALB/12w + PAIR	NP	+	*E. granulosus* G1

^1^ Country or region of origin of the patient or his/her ancestors; ^2^ IHA = indirect hemagglutination titer (cut-off titer 1:32); ^3^ WB = Western blot; ^4^ / = data not available; ^5^ M = male; ^6^ ALB = Albendazole; ^7^ Albendazole therapy duration in weeks; ^8^ PAIR = puncture, aspiration, injection of protoscolicide, reaspiration; ^9^ F = female; ^10^ N Macedonia = North Macedonia; ^11^ BiH = Bosnia and Herzegovina; ^12^ NP = not performed; ^13^ W Balkans = Western Balkans—the patient was of Albanian ethnicity.

**Table 2:** Primer pairs used in the study.

Gene	Primer Name	Sequence (5′–3′)	Tm ^1^ (°C)	Fragment	Reference
				Length (bp ^2^)	
*cox1*	EgCOI1 ^3^	TTTTTTGGCCATCCTGAGGTTTAT	56	444	[38]
	EgCOI2 ^3^	TAACGACATAACATAATGAAAATG			
*nad1*	First PCR:				
	nad1-F	TGGAACTCAGTTTGAGCTTTACTA	54	1239–1242	[54]
	nad1-R	ATATCAAAGTAACCTGCTATGCAG			
	Second PCR:				
	EgNDI1 ^3^	AGTCTCGTAAGGGCCCTAACA	56	530	[38]
	EgNDI2 ^3^	CCCGCTGACCAACTCTCTTTC			
*nad5*	NAD5f ^3^	GCCCCIACICCAGTIAGTTCT	50	297	[55]
	NAD5r ^3^	AAIACACTTAGAIACICCATGACT			
*rrnS*-1	rrnS-F ^3^	AGCCAGGTCGGTTCTTATCTATTG	61	772–781	[56]
	rrnS-R ^3^	CGAGGGTGACGGGCGGTGTGTAC			
*rrnS*-2	Ech12Sf ^3^	AAAIGGTTTGGCAGTGAGIGA	55	285–286	[55]
	Cest12Sr ^3^	GCGGTGTGTACITGAGITAAAC			

^1^ Tm = annealing temperature; ^2^ bp = base pairs; ^3^ primers used also for the sequencing.
